# Epidemiology of measles during the COVID-19 pandemic, a description of the surveillance data, 29 EU/EEA countries and the United Kingdom, January to May 2020

**DOI:** 10.2807/1560-7917.ES.2020.25.31.2001390

**Published:** 2020-08-06

**Authors:** Nathalie Nicolay, Grazina Mirinaviciute, Thomas Mollet, Lucia Pastore Celentano, Sabrina Bacci

**Affiliations:** 1 European Centre for Disease Prevention and Control (ECDC), Stockholm, Sweden

**Keywords:** measles, epidemics, public health surveillance, COVID-19

## Abstract

The number of measles cases declined in European Union/European Economic Area countries and the United Kingdom in 2020. Reported cases to The European Centre for Disease Prevention and Control decreased from 710 to 54 between January and May. Epidemic intelligence screening observed a similar trend. Under-diagnoses and under-reporting during the coronavirus disease (COVID-19) pandemic should be ruled out before concluding reduced measles circulation is because of social distancing and any community control measures taken to control COVID-19.

## Background

From 1 January 2010 to 31 December 2019, European Union/European Economic Area (EU/EEA) countries observed a high level of measles transmission, with 148,279 cases reported to The European Centre for Disease Prevention and Control (ECDC). Major epidemics occurred in 2010 to 2012 and 2017 to 2019 [1]. In 2019, 13,200 cases were reported to ECDC [2]. Immunity gaps and suboptimal vaccination rates in most countries were main factors associated with the last epidemic waves [1,2].

We discuss in this study how the epidemiology and the surveillance of measles in the EU/EEA countries and the United Kingdom (UK) has evolved during the coronavirus disease (COVID-19) pandemic, which was declared on 11 March 2020.

## Data sources

All data presented cover the period 1 January 2010 to 31 May 2020.

Routine monthly indicator-based surveillance data refers to descriptive analysis on confirmed, probable, possible [3] and those classified as unknown cases [2] reported to the European Centre for Disease Prevention and Control (ECDC) by 29 EU/EEA countries and the UK as of 27 June 2020, and retrieved from The European Surveillance System (TESSy) on 30 June 2020. Data may be consolidated in the next months because of delays in case-based reporting to TESSy. The cases reported to TESSy are presented in this report by ‘month of statistics’, which derives from a date chosen by the country for reporting purposes. This date may indicate onset of disease, date of diagnosis, date of notification or date of laboratory confirmation, depending on reporting practices in the respective countries.

Event-based surveillance or epidemic intelligence (EI) data refers to regularly updated measles cases, deaths and outbreaks detected through publicly available official sources, e.g. websites of ministries of health, national institutes of public health, etc. Additional screening of media sources is also performed for the monitoring of measles threats. These data are supplemental to the TESSy data. EI detection aims at identifying cases up to the day of the screening and it is performed approximately 10 to 15 days after the deadline of reporting to TESSy, which, on the other hand, refers to the cases up to the end of the previous month of when the data is collected. The cases detected by EI are presented by ‘month of detection’, derived from the date cases are identified by EI at ECDC. Month of detection and month of statistics do not necessarily match.

We defined the measles peak season as the period between January to May.

## Results

**Figure 1:**
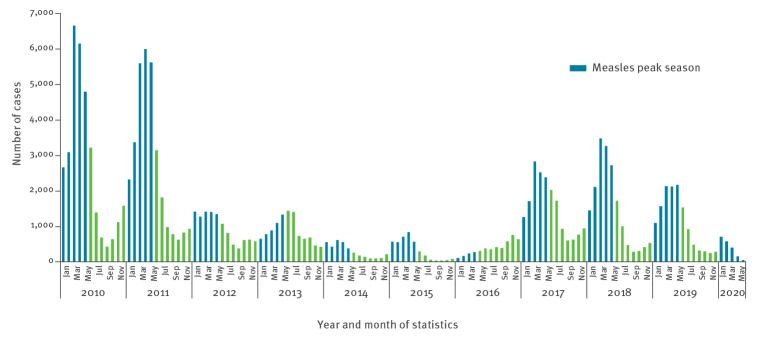
Number of measles cases^a^ reported to TESSy (ECDC) by month of statistics and year, 29 EU/EEA countries and the UK, 1 January 2010–31 May 2020 (n = 150,188 cases, month of reporting was missing for eight cases)

ECDC: European Centre for Disease Prevention and Control; EU/EEA: the European Union/European Economic Area; TESSy: The European Surveillance System; UK: United Kingdom.

^a^ All confirmed, probable, possible based on definitions found in [3], as well as cases with unknown status.

**Figure 2:**
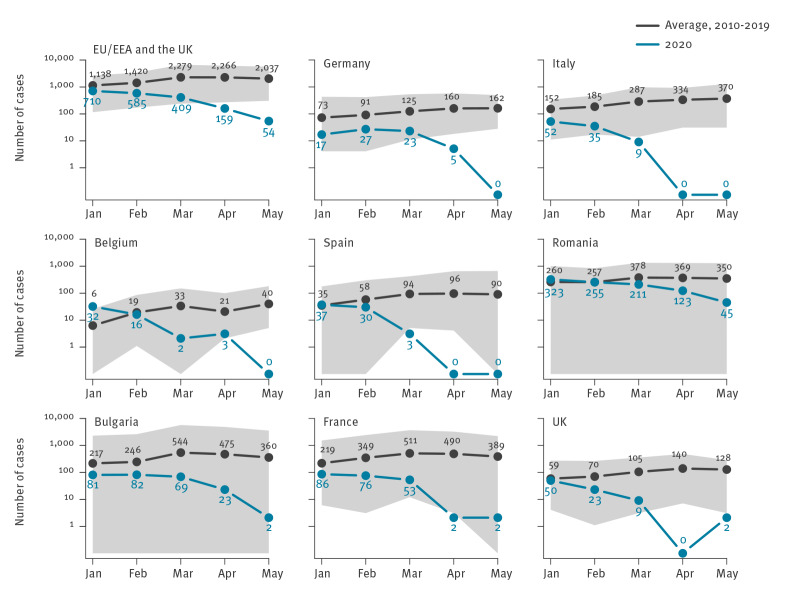
Average number of measles cases^a^ in 2010–2019 and number of measles cases^a^ in 2020 reported to TESSy (ECDC) by month of statistics during the January to May measles season peaks, EU/EEA and the UK and selected countries^b^

ECDC: European Centre for Disease Prevention and Control; EU/EEA: European Union/European Economic Area; TESSy: The European Surveillance System; UK: United Kingdom.

^a^ All confirmed, probable, possible based on definitions found in [3], as well as those cases with unknown status.

^b^ Countries reporting the highest number of cases between 1 January 2020 and 31 May 2020.

Between 1 January 2020 and 31 May 2020, 1,917 measles cases (1,246 confirmed, 390 probable, 276 possible and five unknown) were reported to ECDC. The median age of cases was 7 years and mean age was 14 years (interquartile range (IQR) 1–25). Since January 2010, it was the second lowest number of cases ever reported during a measles peak season (1,104 cases between January to May 2016) (Figure 1). And for the first time, the number of reported cases in 2020 sharply decreased between January (n = 710) and May (n = 54) (Figure 1, Table 1). Among countries that reported the highest total number of cases between January 2020 and May 2020 (Belgium, Bulgaria, Germany, Spain, France, Italy, Romania and the UK), the number of cases fell month by month, contrary to the typically observed increasing trend during these months. Furthermore, in these countries, the number of cases in May 2020 was the lowest ever reached since January 2010 (Figure 1 and 2). Countries did not report imported cases in April and May compared with 6% (35/560, cases with unknown imported status = 150), 5% (22/442, unknown = 143) and 2% (6/313, unknown = 93) of them in January, February and March 2020. From March to June 2020, all 30 countries ensured continuity in the reporting of measles to ECDC on a monthly basis and with maximum delay of 2 days after the deadline.

**Table 1:** Number of measles cases^a^ reported to TESSy (ECDC) by month of statistics and country, 29 EU/EEA countries and the UK, 1 January 2020–31 May 2020 (n = 1,917)

Country	Month (2020)					Total
	January	February	March	April	May	
Austria	2	7	16	0	0	**25**
Belgium	32	16	2	3	0	**53**
Bulgaria	81	82	69	23	2	**257**
Croatia	0	0	0	0	0	**0**
Cyprus	0	1	0	0	0	**1**
Czech Republic	0	3	0	0	0	**3**
Denmark	4	0	0	0	0	**4**
Estonia	0	0	0	0	0	**0**
Germany	17	27	23	5	0	**72**
Greece	1	0	1	0	0	**2**
Finland	2	2	0	0	0	**4**
France	86	76	53	2	2	**219**
Hungary	0	0	0	0	0	**0**
Iceland	0	0	0	0	0	**0**
Ireland	1	12	4	1	0	**18**
Italy	52	35	9	0	0	**96**
Latvia	0	0	0	0	0	**0**
Lithuania	1	0	1	0	0	**2**
Luxembourg	0	0	0	0	0	**0**
Malta	0	2	0	0	0	**2**
Netherlands	1	1	0	0	0	**2**
Norway	3	1	0	0	0	**4**
Poland	6	6	8	2	1	**23**
Portugal	4	2	0	0	2	**8**
Romania	323	255	211	123	45	**957**
Slovenia	5	1	0	0	0	**6**
Slovakia	0	0	0	0	0	**0**
Spain	37	30	3	0	0	**70**
Sweden	2	3	0	0	0	**5**
UK	50	23	9	0	2	**84**
**EU/EEA and the UK**	**710**	**585**	**409**	**159**	**54**	**1,917**

ECDC: European Centre for Disease Prevention and Control; EU/EEA: European Union/European Economic Area; TESSy: The European Surveillance System; UK: United Kingdom.

^a^ All confirmed, probable, possible based on definitions found in [3], as well as those cases with unknown status.

The grey area represents the minimum and the maximum number of cases reported over the 2010–2019 period.

In the same period, January to May, EI identified reports of 2,040 measles cases in 29 EU/EEA countries and the UK (Table 2). After a slight increase observed between January (n = 313) and February (n = 876), monthly data showed a decrease to 292 in March and 253 cases in April 2020, followed by a slight upsurge in May (n = 306). Over the study period, EI screening captured slightly more cases that those reported into TESSy for Romania (n = 1,092) and Spain (n = 82).

**Table 2:** Number of measles cases^a^ identified by Epidemic Intelligence at ECDC by country and by month of detection, 29 EU/EEA countries and the UK, 1 January 2020–31 May 2020 (n = 2,040)

Country	Month (2020)					Total
	January	February	March	April	May	
Austria	1	2	0	21	1	**25**
Belgium	37	11	0	0	0	**48**
Bulgaria	13	147	73	20	4	**257**
Croatia	0	0	0	0	0	**0**
Cyprus	0	0	0	1	0	**1**
Czech Republic	0	3	0	0	0	**3**
Denmark	0	0	4	0	0	**4**
Estonia	0	0	0	0	0	**0**
Finland	0	0	4	0	0	**4**
France	86	75	57	0	0	**218**
Germany	0	27	32	0	0	**59**
Greece	0	1	1	0	0	**2**
Hungary	1	0	0	0	0	**1**
Iceland	0	0	0	0	0	**0**
Ireland	0	13	2	0	0	**15**
Italy	52	34	9	0	0	**95**
Latvia	0	0	0	0	0	**0**
Lithuania	1	0	1	0	0	**2**
Luxembourg	0	0	0	0	0	**0**
Malta	0	2	0	0	0	**2**
Netherlands	0	1	1	0	0	**2**
Norway	3	1	0	0	0	**4**
Poland	2	11	5	3	2	**23**
Portugal	5	1	0	0	0	**6**
Romania	88	524	140	41	299	**1,092**
Slovenia	5	1	0	0	0	**6**
Slovakia	0	0	0	0	0	**0**
Spain	17	22	24	24	0	**87**
Sweden	2	0	0	3	0	**5**
UK	0	0	79	0	0	**79**
**EU/EEA and the UK**	**313**	**876**	**292**	**253**	**306**	**2,040**

ECDC: European Centre for Disease Prevention and Control; EU/EEA: European Union/European Economic Area; UK: United Kingdom.

^a ^As per country’s case definition.

## Discussion

Measles is an acute, highly infectious illness caused by Measles morbillivirus. It is transmitted through airborne respiratory droplets, or by direct contact with nasal and throat secretions of infected individuals [1]. In temperate climates, the illness occurs in a seasonal pattern with cases progressively increasing in winter and peaking in spring. After a large outbreak, the inter-epidemic period varies depending on measles vaccination coverage and the number of susceptible individuals accumulated in each new birth cohort [1].

Since 2011, ECDC has been coordinating measles surveillance at the EU/EEA and UK level following the transfer of the European surveillance network for vaccine preventable diseases (EUVAC.NET), which started collecting data in 1999 [4]. All countries submit measles surveillance data on a monthly basis to TESSy, which is hosted by ECDC. Data are shared with the World Health Organization Regional Office for Europe (WHO/Europe) as part of the measles surveillance of the WHO European Region [5]. In addition, the EI group at ECDC ensures a comprehensive monitoring of the epidemiology of measles by systematically screening various sources of measles information, e.g. number of cases, deaths and measles related events, each month. Results of EI screening are regularly published in communicable disease threat reports (CDTR) [6]. The two systems, EI detection and routine surveillance, should be seen as complementary to each other [7]. Differences in the number of cases captured by the two systems may reflect some delays in data reporting to TESSy (which has been known to occur when large outbreaks are ongoing, as for example, in Romania [8]) and/or differences depending on which month cases are accounted for and/or under-reporting.

In 2020, we observed an unexpected break in the measles outbreak dynamic. For the first time since the implementation of EU/EEA and UK surveillance, there was a sharp decrease in the number of measles cases during the season when the virus usually circulates widely [9]. This finding is also supported by the EI data [6]. By comparison, the last rapid risk assessment published by ECDC in May 2019 highlighted the high risk of sustained measles outbreaks in EU/EEA countries [1]. The COVID-19 pandemic probably had an impact on the epidemiology of measles at the beginning of 2020 in several ways.

First, measles shares the same routes of transmission as COVID-19 [10]. Its reproductive number (R_0_) is much higher than most transmissible diseases [1]. Therefore, any control measures applied to COVID-19 may also effect measles incidence. Confinement strategies were widely implemented in EU/EEA and the UK, including primary and/or secondary school closures in all countries and stay-at-home orders in 18 countries [11], which therefore impact the R_0_. School closures in the past have allowed for the reduction of measles transmission in school-aged children [12] and the control of other close-contact infectious diseases, such as influenza [13,14]. The reduction of social contacts probably had an impact on measles transmission. More in depth analyses are required to describe to what extent the epidemiology of measles has evolved in terms of age groups affected and impact by countries depending on the control measures taken.

Second, the pandemic has been responsible for healthcare services disruptions in hospitals and primary healthcare settings that may have impaired access to care. With hospitals overwhelmed with the inflow of COVID-19 patients and needing reorganisation, clinical practices were also challenged. Telemedicine expanded rapidly among general practitioners and paediatricians in response to the pandemic and the need to adopt social distancing measures [15,16]. However, there is little data on the quality of telemedicine consultation for the aetiological diagnosis of rash diseases [17], especially in low prevalence settings [18]. We cannot rule out that measles was misdiagnosed or that patients with eruptive disease sought less medical attention since the beginning of the pandemic.

Third, in the pre-pandemic era and in some settings, the incidence of measles has been described as being underestimated because of under-reporting [19] even when notification was mandatory [20]. The pandemic may have worsened under-reporting. During measles outbreaks, physicians are reminded of their notification duties through increased measles awareness by public health authorities and physicians’ associations as well as intensified media coverage. Such communication may have been impaired by the COVID-19 pandemic in a number of countries. Physicians may also have considered the notification of sporadic measles cases less of a priority.

Finally, the pandemic is a demanding and a challenging period for public health services. It required massive adjustments in national and local surveillance systems, e.g. influenza surveillance [21]. EU/EEA countries and the UK have shown, since the beginning of the pandemic, great commitment to international surveillance. All countries ensured timely measles reporting from the national level to ECDC. Elimination of measles was a priority in the European Vaccine Action Plan 2015–2020 [22]. A strong surveillance system is one of the four pillars of the WHO/Europe Regional Strategy for measles and rubella elimination [23]. However, the challenges observed at regional or local level may have compromised the ability to describe the real epidemiology of measles, either because of under-reporting or delay in reporting. Frontline public health officers who coordinated the surveillance of infectious diseases in the field may have been overwhelmed by the COVID-19 workload to the detriment of other monitoring systems.

During the COVID-19 pandemic, measles vaccination programme continuity remains an absolute priority [24] in order to ensure high level of vaccination coverage and to avoid any immunity gap. In parallel, we need to ensure that surveillance systems do not collapse and that the decreasing trend in the number of measles cases routinely reported to TESSy reflects the true epidemiology of the disease. EI data collection from publicly available official sources, as well as through media, will continue to be extremely valuable to interpreting the trend and detecting measles clusters. However, it also heavily depends on the diagnostic and reporting capacity of the countries. Continuous monitoring in the coming months will help to better identify the root of this phenomenon, in particular any resurgence of cases once containment measures are lifted. Under-diagnoses and under-reporting during the coronavirus disease (COVID-19) pandemic should be ruled out before concluding reduced measles circulation is because of social distancing measures and any community control measures taken to control COVID-19.
